# Bacterial indole‐3‐butyric acid promotes fish PUFA synthesis

**DOI:** 10.1002/imo2.70136

**Published:** 2026-08-26

**Authors:** Ruojing Li, Biao Yuan, Xin Yi, Jieyan Lin, Bi Huang, Ermei Yin, Jiayu Zhou, Qianwen Min, Meiqi Lv, Bo Zhang, Na Zhao

**Affiliations:** ^1^ Southern Marine Science and Engineering Guangdong Laboratory (Zhanjiang) Zhanjiang China

**Keywords:** gut microbiota, IBA, lipid metabolism, PPARγ, PUFA

## Abstract

Marine fish are paradoxically rich in polyunsaturated fatty acids (PUFAs) despite limited endogenous biosynthetic capacity, suggesting a critical contribution from the gut microbiota. Here, we first characterized the gut microbiota of *Seriola dumerili*, a PUFA‐rich marine fish, functionally enriched in the biosynthesis of unsaturated fatty acids. Further functional validation was performed using zebrafish as a model. Strain screening revealed the *Bacillus thuringiensis* strain Sd_h10 exhibited potent lipid utilization capacity *in vitro* and in zebrafish on a high‐fat diet, and remodeled transcription of lipid‐related pathways to promote lipid accumulation and PUFA biosynthesis. Notably, indole‐3‐butyric acid (IBA), a major metabolite of Sd_h10, was found in the intestinal tissue of *S. dumerili* and regulated peroxisome proliferator‐activated receptor gamma (PPARγ), mimicking the effects of the PPARγ agonist rosiglitazone in zebrafish. Long‐term IBA administration in conventional zebrafish increased body weight gain, hepatic lipid accumulation, and muscle docosahexaenoate (DHA) content, accompanied by upregulation of PPARγ downstream target genes, including *dgat2*, *fads6*, and *elovl6l*. Simultaneously, IBA reshaped the intestinal microbiota, depleting *Burkholderiaceae* while enriching *Acidovorax* abundance, accompanied by enrichment of functions related to fatty acid uptake, lipid utilization, and unsaturated fatty acid modification. These findings support a potential role for the microbiota‐derived metabolite IBA in modulating PPARγ‐associated signaling pathways linked to host DHA biosynthesis and accumulation. Our study provides additional evidence supporting microbiota‐host lipid metabolic interactions and highlights the potential application of probiotic‐associated microbial metabolites for improving lipid nutritional quality in marine fish.

## Highlights

- A gut‐derived *Bacillus* metabolite, indole‐3‐butyric acid (IBA), promotes polyunsaturated fatty acid (PUFA) biosynthesis in fish.
- IBA interacts with PPARγ and regulates lipid metabolism‐related signaling pathways associated with PUFA accumulation.
- Microbiota‐derived indole metabolites represent potential regulators linking the gut microbiota to host lipid metabolism.

## Graphical abstract

Polyunsaturated fatty acids (PUFAs) are essential nutrients in aquatic products, yet the contribution of gut microbiota‐derived metabolites to PUFA biosynthesis remains largely unknown. In this study, a gut‐derived *Bacillus* strain was identified to enhance PUFA accumulation via indole‐3‐butyric acid (IBA) production. Integrated microbiological, metabolomic, functional, and molecular docking‐based analyses demonstrated that IBA interacted with PPARγ and regulated lipid metabolism‐related signaling pathways, thereby promoting PUFA biosynthesis in fish. These findings reveal a microbiota‐metabolite‐host regulatory axis and identify microbial indole metabolites as potential regulators of host lipid metabolism.

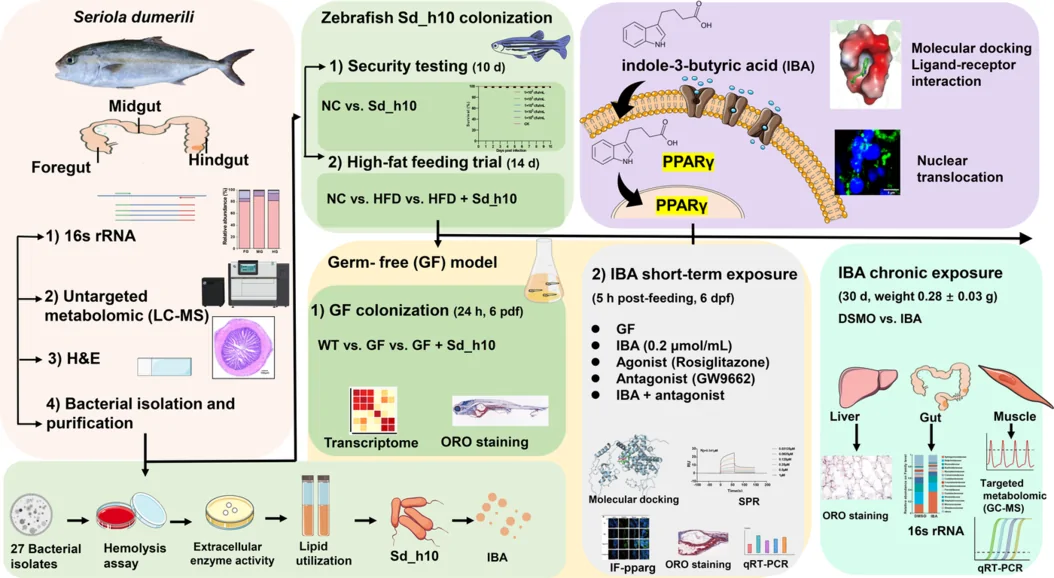

## 1 INTRODUCTION

Polyunsaturated fatty acids (PUFAs) are essential nutrients supporting vertebrate growth and development, and fish are a primary dietary source of many PUFAs [1, 2]. Despite the high tissue PUFA content observed in many marine fish, their endogenous capacity for *de novo* PUFA biosynthesis is relatively limited and species‐dependent, creating a paradox regarding how these animals maintain such elevated PUFA levels [3, 4]. Beyond host genetics and dietary intake, the gut microbiota has emerged as a key modulator of lipid and PUFA metabolism [5, 6, 7]. Gut microbiota plays a pivotal role in regulating lipid metabolism in fish through multiple mechanisms. First, intestinal microbes directly participate in lipid digestion and absorption by secreting lipases, which hydrolyze dietary lipids into free fatty acids (FFAs) and monoglycerides [8, 9]. In addition, gut microbiota‐derived metabolites, specifically short‐chain fatty acids (SCFAs) and secondary bile acids, act as critical signaling mediators that integrate microbial activity with host lipid metabolism. SCFAs generated through microbial fermentation not only fuel intestinal epithelial cells but also modulate signaling pathways involving PPARs and G‐protein‐coupled receptors, thereby influencing host insulin sensitivity and lipid metabolic gene expression [10, 11, 12]. Similarly, microbiota‐modified bile acids regulate host lipid metabolism through receptor‐mediated signaling pathways [13, 14, 15]. Among these pathways, PPARγ is increasingly recognized as an important regulator of fatty acid remodeling and PUFA‐associated lipid metabolism in teleosts [16, 17].

Compared with SCFAs and bile acids, indole derivatives are structurally distinct aromatic scaffolds that may enable interactions with nuclear receptor‐associated signaling pathways. Medicinal chemistry and structural studies have shown that indole‐containing compounds can interact with nuclear receptors such as PPARγ, and several synthetic indole derivatives have been developed as partial PPARγ agonists capable of modulating lipid and glucose homeostasis [18]. Recent evidence further suggests that tryptophan‐derived indole metabolites, including indole‐3‐acetic acid (IAA), participate in host metabolic regulation and influence hepatic lipid metabolism and insulin sensitivity in mammalian models [15, 16, 17]. These findings highlight indole metabolites as important microbiota–host signaling molecules. However, their regulatory roles in fish lipid metabolism, particularly in PUFA‐associated pathways, remain poorly understood. Moreover, the biological functions of specific indole metabolites, especially indole‐3‐butyric acid (IBA), have been rarely explored in aquatic animals.

Nevertheless, whether gut microbiota‐derived indole metabolites participate in the regulation of PUFA biosynthesis in fish remains largely unknown. In particular, it is unclear whether these metabolites modulate host lipid metabolic remodeling through PPARγ‐associated signaling pathways. Previous studies have mainly focused on microbial fatty acids, bile acids, and related derivatives as modulators of host lipid metabolism through nuclear receptor‐associated pathways [18, 19]. In contrast, the contribution of indole‐derived metabolites to PUFA metabolic remodeling has received far less attention, particularly in marine teleosts. IBA is a well‐characterized signaling molecule in plants [20]. Yet, its biological role in vertebrates, especially in lipid metabolism and PUFA accumulation, remains poorly understood. Given the central role of PPARγ in PUFA‐associated lipid metabolic remodeling, microbiota‐derived indole metabolites may represent an unrecognized regulatory axis linking gut microbial activity to host PUFA biosynthesis. Accordingly, we hypothesized that intestinal microbes regulate host PUFA biosynthesis through the production of indole‐derivative metabolites that modulate host PPARγ‐associated signaling pathways. To test this hypothesis, we profiled the gut microbiota and metabolome of the PUFA‐rich marine fish *S. dumerili* [21], isolated key bacterial strains, and identified an indole derivative‐producing *Bacillus thuringiensis* (Sd_h10). Germ‐free and conventional zebrafish were utilized as model organisms to verify that its primary metabolite IBA exhibits PPARγ‐modulating activity and regulates PUFA biosynthesis. This integrated framework supports a potential microbiota‐indole derivative‐PPARγ regulatory axis that expands current understanding of microbiota‐associated lipid metabolic regulation and provides a basis for nutritional strategies of marine fish.

## 2 RESULTS

### High‐PUFA phenotype of *S. dumerili* muscle

Muscle PUFA content in *S. dumerili* was among the highest across the collected marine species [22, 23], and second only to *Mugil cephalus* and higher than in *Acanthopagrus schlegelii* and *Larimichthys crocea* (Figure 1A).

**Figure 1:**
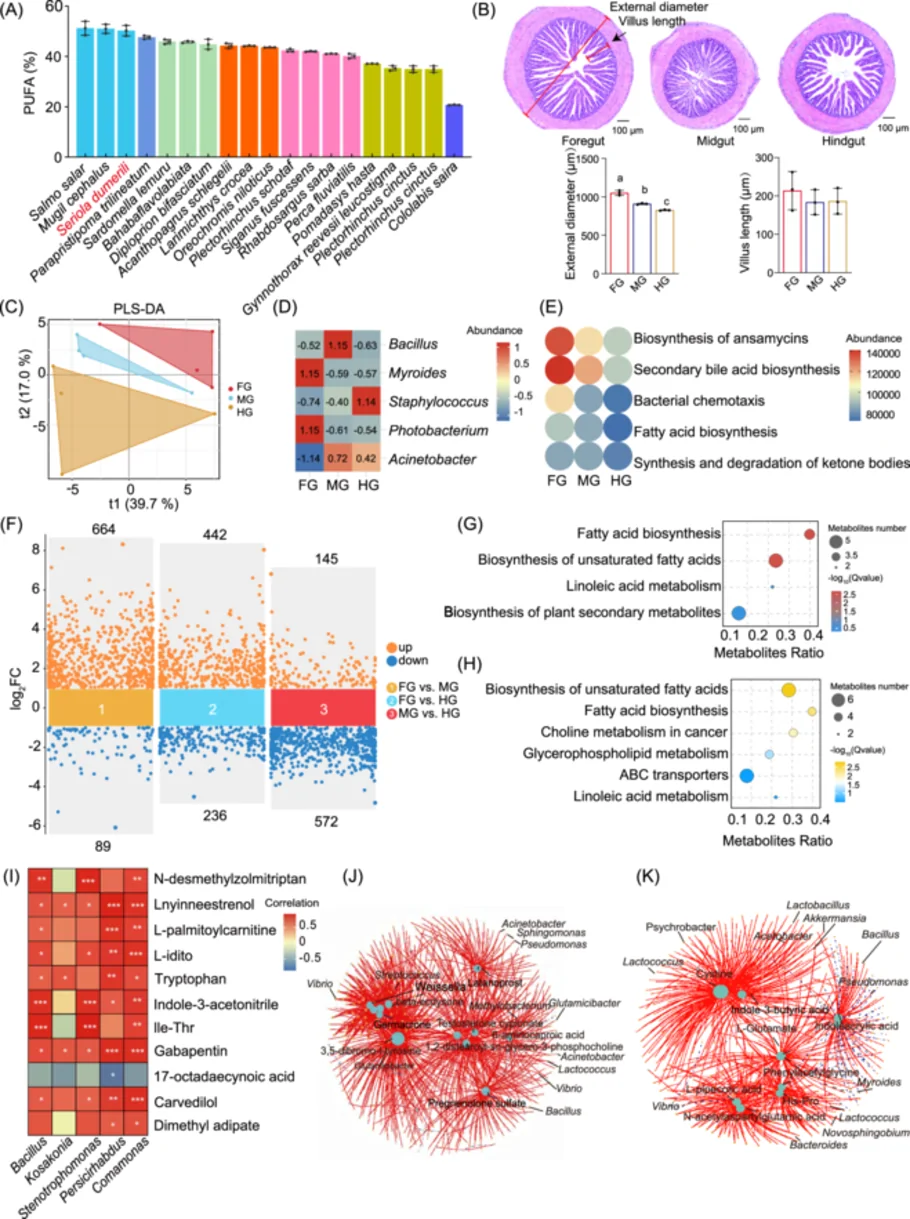
Segmental differences in gut microbiota composition contrast with conserved lipid metabolic functions in *S. dumerili*. (A) Aggregated data on muscles PUFA content (%) in marine fish species from published literature. *S. dumerili* is highlighted in red. Data were normalized to the percentage of total fatty acids to ensure comparability across studies. (B) Histological structure of *S. dumerili* different intestinal segments under H&E staining. Bar charts show the external diameter and villus length. FG, foregut; MG, midgut; HG, hindgut. Values with different small letter indicated significant difference (*p* < 0.05), *n* = 3 per group. (C) Partial least squares discriminant analysis (PLS‐DA) of microbial community structure at the OTU level across intestinal segments. (D) Relative abundance of dominant genera in FG, MG, and HG groups. Data subjected to *Z*‐Score normalization based on row data. (E) Heatmap of the relative abundance of metabolic pathways (KEGG level 3, top 5) predicted through PICRUSt2 functional analysis of 73 core microbes present in the FG, MG, and HG. (F) Scatter plots showing differential metabolites between FG, MG, and HG. Orange and blue dots indicate significantly upregulated and downregulated metabolites, respectively (FDR < 0.05). (G) KEGG enrichment analysis of FG‐upregulated metabolites compared with HG (*Q*‐value < 0.05). (H) KEGG enrichment analysis of MG‐upregulated metabolites compared with HG (*Q*‐value < 0.05). (I) Pearson correlation heatmap between metabolites and gut microbial genera (top 10 correlation coefficients), *p < 0.05, **p < 0.01, ***p < 0.001. Co‐occurrence networks depicting correlations between intestinal microorganisms and lipid metabolites and their derivatives (J) and organic acids (K). Blue and yellow dots represent metabolites and bacterial genera, respectively. Red edges denote positive correlations and blue edges denote negative correlations (adjusted *p* < 0.05). H&E, hematoxylin and eosin; PUFA, polyunsaturated fatty acid.

### Intestinal characteristics of the high‐PUFA fish *S. dumerili*

Histological examination revealed clear regional specialization along the intestine of *S. dumerili* (Figure 1B). The foregut (FG) exhibited the largest external diameter (1082.15 ± 24.40 μm), followed by the hindgut (HG, 933.40 ± 9.49 μm) and midgut (MG, 848.96 ± 6.00 μm; *p* < 0.05). Villus length gradually declined from anterior to posterior regions (FG: 216.32 ± 59.52 μm; MG: 181.41 ± 45.88 μm; HG: 185.76 ± 49.03 μm), consistent with the overall reduction in absorptive surface area. These results confirmed that *S. dumerili* was a high‐PUFA species with pronounced intestinal differentiation.

### Intestinal microbiota exhibits compositional differences but conserved lipid metabolic functions across segments

A total of 3296 operational taxonomic units (OTUs) were annotated to 25 phyla and 292 genera. Alpha diversity, measured by Chao and ACE indices, was highest in FG, followed by HG (Table S1). Multivariate analyses, including partial least squares discriminant analysis (PLS‐DA, Figure 1C) and analysis of similarities (ANOSIM, Figure S1A), confirmed significant compositional differences among intestinal segments of *S. dumerili* (*p* < 0.05). At the phylum level, *Proteobacteria*, *Bacteroidetes*, and *Firmicutes* dominated all segments, with *Proteobacteria* accounting for over 80% of total abundance. The relative abundance of *Firmicutes* increased progressively from FG to HG (Figure S1B). *Myroides*, *Bacillus*, and *Staphylococcus* had the highest relative abundance in FG, MG, and HG, respectively (Figure 1D). Despite taxonomic variation, KEGG Level 2 functions were highly conserved across regions, with lipid metabolism and amino acid metabolism consistently ranking among the top five categories (Figure S1C).

A total of 73 genera were shared (Figure S1D, Table S2, such as *Acinetobacter* and *Photobacterium*) among the intestinal segments. Functional predictions indicated that these core taxa were particularly enriched in lipid metabolism‐related pathways, namely Secondary bile acid biosynthesis, Fatty acid biosynthesis, and Synthesis and degradation of ketone bodies (Figure 1E). Collectively, while microbial composition varied along the intestinal segments, functional redundancy was still maintained, particularly in pathways related to lipid processing and energy metabolism.

### Intestinal metabolites vary regionally but are dominated by lipid metabolism

A total of 5554 metabolites were detected across the intestinal segments of *S. dumerili*. Orthogonal partial least squares discriminant analysis revealed distinct metabolic profiles among the foregut (FG), midgut (MG), and hindgut (HG) (Figure S2A–C). Lipids and lipid‐like molecules, such as peridinin, tauroursodeoxycholic acid, methyl hexadecanoate, and 7‐keto‐8‐aminopelargonic acid, represented a major portion of the differential metabolites (Figure 1F). Most metabolites enriched in KEGG Level 2 categories were related to lipid and amino acid metabolism in all comparison groups (Figure S2D). Pathway enrichment analyses showed that downregulated differentially expressed metabolites (DEMs) in FG were enriched in vitamin and amino acid metabolism (Figure S3A,B). Compared to HG, the upregulated metabolites in FG and MG were significantly enriched in Fatty acid biosynthesis, Biosynthesis of unsaturated fatty acids, and Linoleic acid metabolism (Figure 1G,H). In contrast, downregulated DEMs in MG were related to Carbohydrate metabolism and Amino acid metabolism (Figure S3C). Metabolomics pathway analysis (MetPA) further indicated that the important metabolic pathways involved in differential metabolites across groups were Purine metabolism, Lysine degradation, Glutathione metabolism, and Steroid biosynthesis (Level 3, Figure S3D).

### Microbial communities closely associated with lipid‐related metabolites

Two‐way orthogonal partial least squares (O2PLS) modeling revealed a strong covariation between microbial community structure and metabolite profiles, with linoleic acid and pterine contributing to community differentiation (Figure S3E). Pearson correlation analysis further showed that bacterial taxa, including *Persicirhabdus*, *Bacillus*, and *Comamonas*, were positively correlated with lipid and lipid‐like molecules (e.g., gabapentin, kirenol, indole‐3‐acetonitrile) (Figure 1I, *p* < 0.05). Network analyses identified distinct co‐occurrence modules between microbes and metabolites. For instance, lipid derivatives including latanoprost, pregnenolone sulfate, and germacrone were correlated with *Acinetobacter*, *Bacillus*, *Vibrio*, and *Lactococcus* (Figure 1J). Organic acid metabolites such as IBA, cystine, and l‐glutamate showed significant correlations with *Novosphingobium* and *Bacteroides* (Figure 1K). Together, these results revealed coordinated microbe‐metabolite crosstalk, implying potential associations between gut microbial composition and the intestinal lipid metabolic environment of *S. dumerili*.

After sequence deduplication, 27 bacterial strains were isolated and identified from the gut of *S. dumerili*. Gram staining revealed 14 Gram‐negative and 13 Gram‐positive strains, and hemolytic assays classified the isolates into α‐hemolytic (8), β‐hemolytic (9), and γ‐hemolytic (9) groups (Figure 2A–C). Among these, strain Sd_X3 only demonstrated sensitivity towards tetracycline, and Sd_H6 was the most sensitive to antibiotics (Table S3). Notably, 7 of 10 *Vibrionales* strains showed hemolytic activity, while most *Bacillales* isolates were non‐hemolytic (Figure 2A). As for extracellular enzyme screening, all 12 tested strains produced protease and caseinase, while 10 strains exhibited amylase activity (Figure 2D). Six isolates (strains Sd_Y15, Sd_IC2, Sd_h5, Sd_h10, Sd_y1, and Sd_y3) displayed lipase activity, and six secreted lecithinase. Each strain demonstrated at least four enzyme activities, highlighting their potential roles in host nutrient digestion. Remarkably, the extracellular products of strain Sd_y3 (*Brachybacterium sp*.) exhibited all seven tested enzymatic functions.

**Figure 2:**
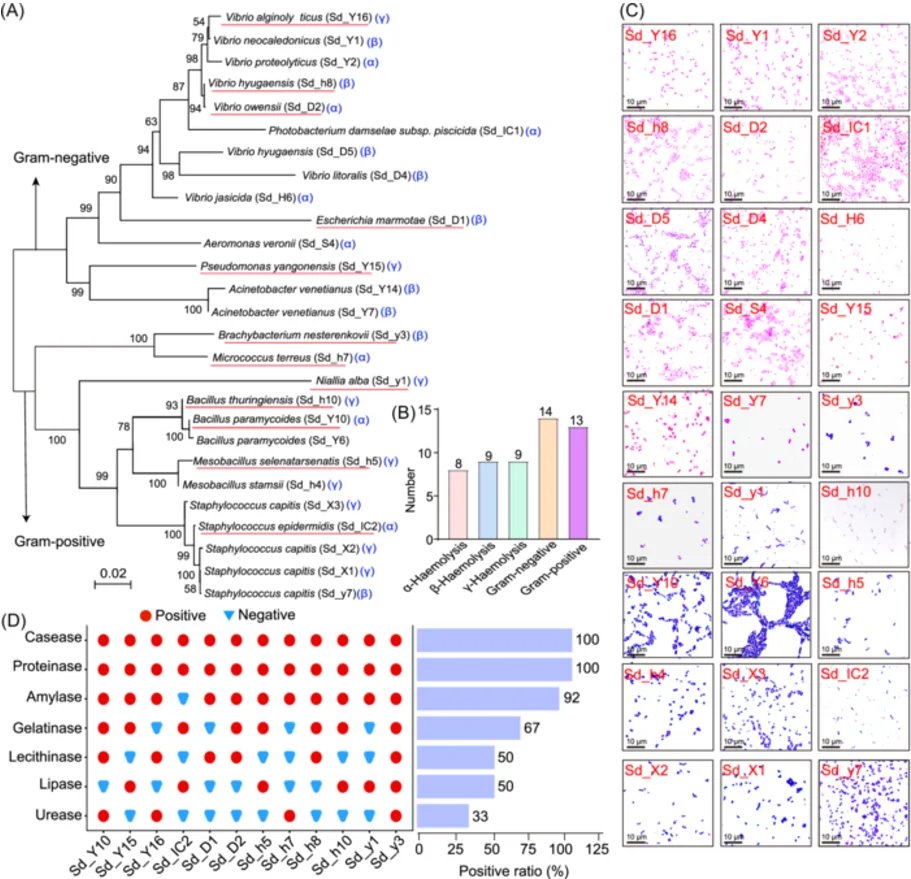
Gut isolates display heterogeneous hemolytic phenotypes but share broad extracellular enzymatic capabilities. (A) Phylogenetic tree of 27 isolates constructed from 16S rRNA gene sequences, with strain names and hemolytic types annotated. Strain names and hemolytic types (*α*, *β*, and *γ*) are indicated in black and blue brackets, respectively. Strains with red underline were selected for extracellular enzyme assays. Scale bar: 0.02 substitutions per site. (B) Separate statistics were made for Gram staining results and hemolytic results, and numbers above bars indicate the count. (C) Gram staining of all isolates showing 14 Gram‐negative and 13 Gram‐positive strains (Gram‐positive for Sd‐y3 and subsequent strains), scale bar = 10 μm. (D) Extracellular products of the 12 isolated strains had both case and protease activities. Red dots indicate positive activity and blue triangles indicate negative activity. Bar plots on the right summarized the proportion of isolates positive for each enzyme.

### Outstanding lipid utilization capacity of Sd_h10

The lipid degradation capacity assay used three representative non‐hemolytic isolates with lipase activity, including *Niallia alba* Sd_y1, *Pseudomonas yangonensis* Sd_Y15, and *Bacillus thuringiensis* Sd_h10. Strain *N. alba* Sd_y1 exhibited negligible growth across all lipid‐supplemented media (fish oil, soybean oil, and paraffin) (Figure S4A–C). Sd_Y15 was capable of growth in all three additives and displayed faster growth in fish oil (Figure S4D–F). In contrast, Sd_h10 achieved the maximum growth rate and biomass yield in fish oil, significantly outperforming the other strains (Figure S4G–I).

In tryptic soy broth (TSB) containing 2%, 8%, or 16% fish oil, Sd_h10 incubation for 36 h led to a marked decline in TGs and concentration‐dependent changes in FFAs (Figure 3A,B). Sd_h10 exhibited growth when cultured in fish oil, while no growth occurred in paraffin (Figure 3C). In Sd_h10 cultures with fish oil as the sole carbon source, TGs decreased significantly after 12 h and plateaued thereafter. FFA peaked at 14 h before gradually declining (Figure 3D). Under high‐fat feeding, zebrafish juveniles in both high‐fat diet (HFD) and high‐fat die + Sd_h10 (HFD + Sd_h10) exhibited lower relative body length growth and body weight gain than the normal diet (NC). However, Sd_h10 colonization partially alleviated the growth‐suppressive effects of the HFD (Figure 3E).

**Figure 3:**
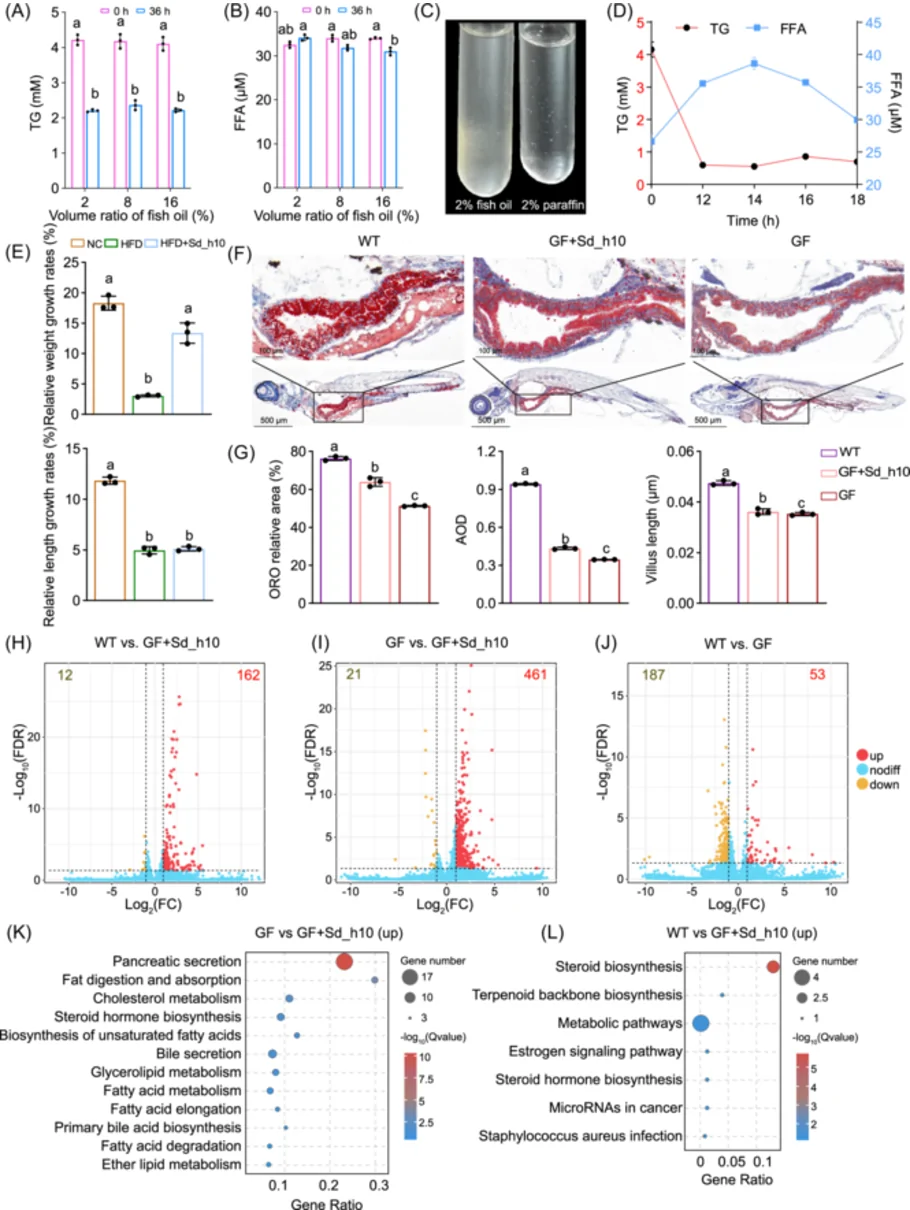
Sd_h10 improves lipid utilization *in vitro* and modulates larval zebrafish lipid accumulation and lipid metabolic transcription. (A, B) Triglyceride (TG) and free fatty acid (FFA) levels in TSB supplemented with 2%, 8%, or 16% (v/v) fish oil after 36 h incubation with Sd_h10. TG and FFA levels decreased in a concentration‐dependent trend. A *t‐*test was performed to analyze the significant differences between 0 h and 36 h. Different lowercase letters indicate significant differences (*p* < 0.05), *n* = 3 per group. (C) Growth of Sd_h10 in fish oil (on the left) or paraffin (on the right) as the sole carbon source. Sd_h10 exhibited robust growth in fish oil, whereas no growth occurred with paraffin. (D) TG and FFA contents in the supernatant of Sd_h10 strain after 18 h incubation in fish oil (2%, v/v) as the sole carbon source. (E) Relative body weight and body length growth rates of zebrafish under NC (normal diet), HFD (high‐fat diet), or HFD + Sd_h10 (high‐fat + Sd_h10) (one‐way ANOVA, *n* = 3 per group). (F) Oil Red O (ORO) staining of zebrafish larvae: WT (wild type), GF + Sd_h10 (germ‐free + Sd_h10), and GF (germ‐free) (one‐way ANOVA, *n* = 3 per group). Upper panels display magnified intestinal segments (scale bar = 100 μm), while lower panels show whole larvae (scale bar = 500 μm). (G) Quantification of ORO staining in zebrafish intestines. Relative stained area, average optical density (AOD) and villus length for WT, GF+ Sd_h10, and GF groups. Significant differences were found among the three groups (one‐way ANOVA, *p* < 0.05, *n* = 3 per group). Volcano plots of differential expressed gene (DEGs) between WT versus GF + Sd_h10 (H), GF versus GF + Sd_h10 (I), and WT versus GF (J). Red and blue dots represent significantly upregulated and downregulated genes, respectively (FDR < 0.05). (K) KEGG enrichment analysis of genes upregulated in GF + Sd_h10 compared with GF, highlighting 12 lipid metabolism‐related pathways. (L) KEGG enrichment of genes upregulated in GF + Sd_h10 versus WT. Pathways associated with lipid metabolism (*Q*‐value < 0.05). Different lowercase letters indicate significant differences (*p* < 0.05).

### Safe colonization of Sd_h10 and its promotion of lipid accumulation in zebrafish

Sd_h10 colonization caused no mortality in zebrafish (Figure S5A). Oil Red O staining (ORO) of zebrafish larvae revealed significant differences in intestinal lipid deposition among treatment groups. WT exhibited maximal lipid deposition, reflected by a larger stained area and elevated average optical density (AOD), while GF (germ‐free) zebrafish displayed minimal staining (Figure 3F,G). Remarkably, the GF + Sd_h10 showed more densely packed red lipid droplets with a stronger staining intensity compared to the GF group (*p* < 0.05). Colonization with Sd_h10 significantly increased both the stained area and AOD in GF zebrafish (*p* < 0.05). WT displayed the longest villus length compared to GF + Sd_h10 and GF (*p* < 0.05), and there were no significant differences between GF + Sd_h10 and GF. These results suggested that Sd_h10 contributed to lipid accumulation in vivo.

### Sd_h10 colonization alters gene expression to regulate lipid metabolic pathways

Principal component analysis (PCA) indicated that the WT, GF, and GF + Sd_h10 were clustered within their respective confidence ellipses (Figure S5B). The volcano plot revealed extensive differential gene expression. For example, compared to WT and GF, the GF + Sd_h10 showed upregulation of genes associated with lipid digestion and absorption (*cel.1*, *cel.2*, *cpb1*, *cpa1*) (Figure 3H,I). However, the GF exhibited downregulation of genes involved in amino acid and carbohydrate metabolism, compared to WT (*rrm2*, *hsp90aa1.1*) (Figure 3J). There were some shared differentially expressed genes (DEGs) among the comparison groups (Figure S5C), with three genes showing differential expression in all three comparison groups (*ces2b*, *cel.1*, and *vwa11*). Compared to GF, genes significantly upregulated in GF + Sd_h10 were enriched in 39 metabolic pathways, including 12 related to lipid metabolism (e.g., fat digestion and absorption, biosynthesis of unsaturated fatty acids, and fatty acid metabolism) (Figure 3K). Compared to WT, upregulated DEGs in the GF + Sd_h10 were enriched in six significantly enriched metabolic pathways, notably steroid biosynthesis and steroid hormone biosynthesis (Figure 3L). Validation by reverse transcription‐quantitative real‐time polymerase chain reaction (qRT‑qPCR) confirmed the RNA‐seq trends with high correlation (*R* ^2^ = 0.91; Figure S5D,E), supporting the reliability of the transcriptomic data.

### Identification of IBA as a microbial indole derivative potentially interacting with PPARγ

Liquid chromatography‐mass spectrometry (LC‐MS) analysis identified several indole‐related metabolites, such as DL‐Tryptophan, indole‐3‐acrylic acid, and IBA, in the spent medium of Sd_h10 and Sd_Y15 (fish oil culture), and gut tissue of *S. dumerili*. Among these, IBA was significantly more abundant than Indole‐3‐acrylic acid in *S. dumerili* gut (Figure 4A), suggesting that microbial IBA production, likely from Sd_h10 or related strains, contributed to the intestinal indole pool. Molecular docking predicted that PPARγ (Figure 4B) could bind to IBA (Figure 4C) with favorable binding energies. The binding pose of IBA (Figure 4D) formed hydrogen bonds with key residues Pro476 and His475 within the activation domain of PPARγ (Figure 4E). IBA exhibited a stronger interaction (−6.17 kcal/mol), forming more hydrophobic interactions and hydrogen bonds with the PPARγ ligand‐binding domain (Figure 4F). In addition, no significant difference was observed in the survival rate of zebrafish between GF and IBA groups (Figure S5F). Collectively, these findings identified IBA as a major microbial metabolite associated with Sd_h10 both in vitro and in the host intestine that might interact with PPARγ. Further tests using surface plasmon resonance (SPR) verified that IBA could effectively bind to PPARγ with a dissociation constant *K*_D_ = 0.541 μM (Figure 4G).

**Figure 4:**
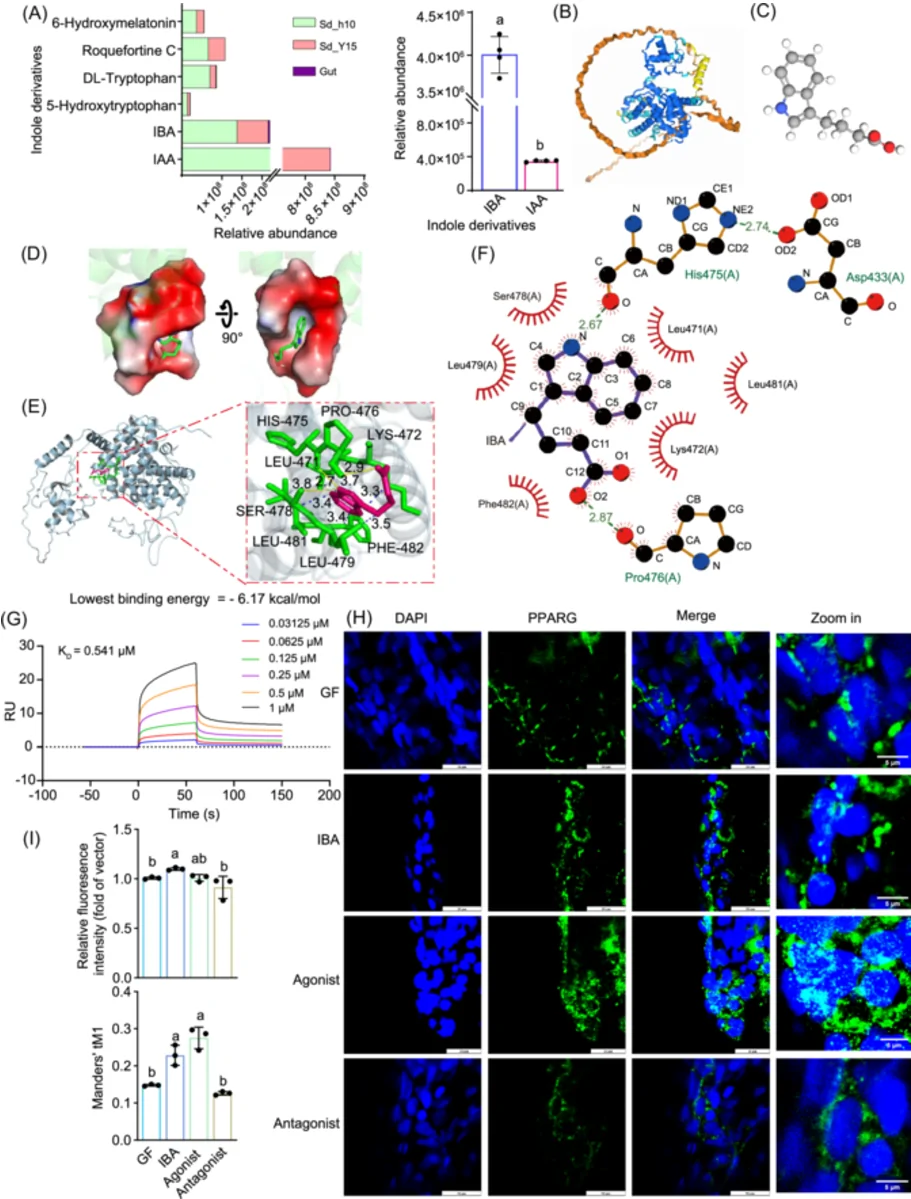
IBA binds to the PPARγ receptor and mimics the effect of PPARγ agonist in nuclear translocation. (A) Indole derivatives levels in bacterial cultures and fish intestine. Relative abundance of six indole derivatives in the spent medium of two strains (Sd_h10 and Sd_Y15), and in the intestine of *S. dumerili*. Comparison of indole‐3‐butyric (IBA) and indole‐3‐acetic acid (IAA) relative abundance in intestinal samples of *S. dumerili*. Different lowercase letters indicate significant differences (*p* < 0.05), *n* = 3 per group. (B) 3D structure of zebrafish peroxisome proliferator‐activated receptor gamma (PPARγ), which was predicted using AlphaFold3. (C) Molecular structure of IBA. The ball‐and‐stick model illustrates the atomic composition, where colors correspond to atoms: gray (carbon, C), white (hydrogen, H), red (oxygen, O), and blue (nitrogen, N). (D) Molecular docking model showing the binding pose of IBA within the PPARγ ligand‐binding pocket. (E) 3D results of molecular docking between PPARγ and IBA, while the panel on the right shows a magnified view of the binding region. The red stick is the conformation of IBA at the time of docking, while the green stick is the amino acid residues interacting with IBA in the PPARγ protein. Hydrogen bonds (yellow solid lines), hydrophobic interactions (blue dashed lines), and numerical value indicating the distance between the sites of interactions (Å). (F) 2D results of molecular docking of IBA and PPARγ, showing hydrogen bonds (green dashed lines) and corresponding bond lengths (Å). Red semicircular arcs indicate hydrophobic interactions between IBA and surrounding residues (Leu471, Leu481, Phe482, etc.). Atoms are colored by element: black = carbon, blue = nitrogen, red = oxygen. (G) Binding affinity measurement of IBA to PPARγ by Surface Plasmon Resonance (SPR). PPARγ protein was immobilized on a CM5 sensor chip, and IBA was injected as the analyte at concentrations of 0.03125 μM−1 μM. The equilibrium dissociation constant is *K*_D_ = 0.541 μM. RU: Resonance Units reflect the change in mass concentration at the sensor surface. (H) Immunofluorescence staining with PPARγ (green), along with DAPI (blue) in the zebrafish larvae. Representative photomicrographs at 40× magnification for PPARγ, scale bar = 20 μm, for the images of PPARγ, DAPI, and Merge, scale bar = 5 μm for zoomed‐in images. (I) Quantification of PPARγ nuclear translocation. Histograms showed PPARγ fluorescence nuclear localization by Manders' tM1 (PPARγ/DAPI) among different treatments (one‐way ANOVA). Relative fluorescence intensity among groups (one‐way ANOVA), representing the mean fluorescence intensity of PPARγ. Different lowercase letters indicate significant differences (*p* < 0.05), *n* = 3 per group.

### Immunofluorescence (IF) staining for PPARγ nuclear translocation

Immunofluorescence staining was performed to examine the PPARγ nuclear translocation in zebrafish larvae. In the GF group, PPARγ was predominantly localized in the cytoplasmic. The IBA and agonist groups showed markedly increased nuclear accumulation of PPARγ, with green fluorescence overlapping DAPI signals, whereas the antagonist group displayed a staining pattern similar to that of the GF group (Figure 4H). Total cellular PPARγ levels were quantified by relative fluorescence intensity. The IBA group showed a slight but significant increase compared to GF (1.08 ± 0.05, *p* < 0.05), with no significant difference between IBA and agonist (*p* > 0.05). The antagonist group exhibited the lowest intensity (0.90 ± 0.04, *p* < 0.05). PPARγ nuclear translocation was quantified by Manders' tM1 coefficient (Figure 4I). The IBA and agonist groups showed the highest tM1 values (0.22 ± 0.03 and 0.28 ± 0.04, respectively; *p* < 0.05), while the antagonist group was significantly lower than all groups (0.12 ± 0.02, *p* < 0.05). These results suggested that IBA partially mimics the effects of agonist in inducing PPARγ nuclear translocation, whereas the antagonist reduces both nuclear entry and total PPARγ protein levels.

### IBA exhibits agonist‐like effects on lipid accumulation and PUFA biosynthesis

IBA treatment produced effects similar to the effects of the PPARγ agonist and opposite to the PPARγ antagonist. Intestinal ORO staining and morphometric analysis revealed that both the agonist and IBA groups exhibited significantly enhanced intestinal lipid deposition and villus elongation compared with the control group, as indicated by increased ORO‐stained areas, AOD values, and villus length (all *p* < 0.05; Figure 5A,B). Concurrently, PUFA levels were elevated in both treatment groups (Figure 5C). At the transcriptional level, IBA and agonist consistently upregulated both *pparγ* (peroxisome proliferator‐activated receptor gamma) and *ins* (insulin) and their downstream genes related to lipid metabolism, including *lipea* (hormone‐sensitive lipase), *dgat2* (diacylglycerol O‐acyltransferase 2), *lpl* (lipoprotein lipase), *cel.1* (carboxyl ester lipase 1), *elovl6l* (elongation of long chain fatty acids like) and *fads6* (fatty acid desaturase 6). Meanwhile, growth‑promoting genes *gh1* (growth hormone 1) and *igf2* (insulin‐like growth factor 2) were also significantly induced (*p* < 0.05). In contrast, IBA and agonist suppressed the expression of *acaca* (acetyl‐CoA carboxylase alpha), *fasn* (fatty acid synthase), and *mstnb* (myostatin b) involved in the production of long‐chain fatty acids and growth inhibition (Figure 5D). Conversely, the antagonist markedly reduced lipid accumulation, PUFA contents, and related gene expression (*p* < 0.05). Importantly, co‐treatment with IBA and the antagonist partially alleviated the inhibitory effects of the antagonist on intestinal lipid accumulation. This was evidenced by significant changes in ORO staining (Figure S6A,B, two‐way ANOVA, partial *η* ^2^ = 0.96), intestinal villus length (Figure S6C, two‐way ANOVA), and PUFA levels (Figure S6D, two‐way ANOVA, partial *η* ^2^ = 0.73) and supported by an interaction effect between IBA and the antagonist (Table S4). The expression of selected PPARγ‐related genes showed variable responses, with some markers being significantly restored while others were not consistently affected (Figure S6E–H). These results suggested that IBA exerts partial effects on antagonist‐induced lipid metabolic suppression.

**Figure 5:**
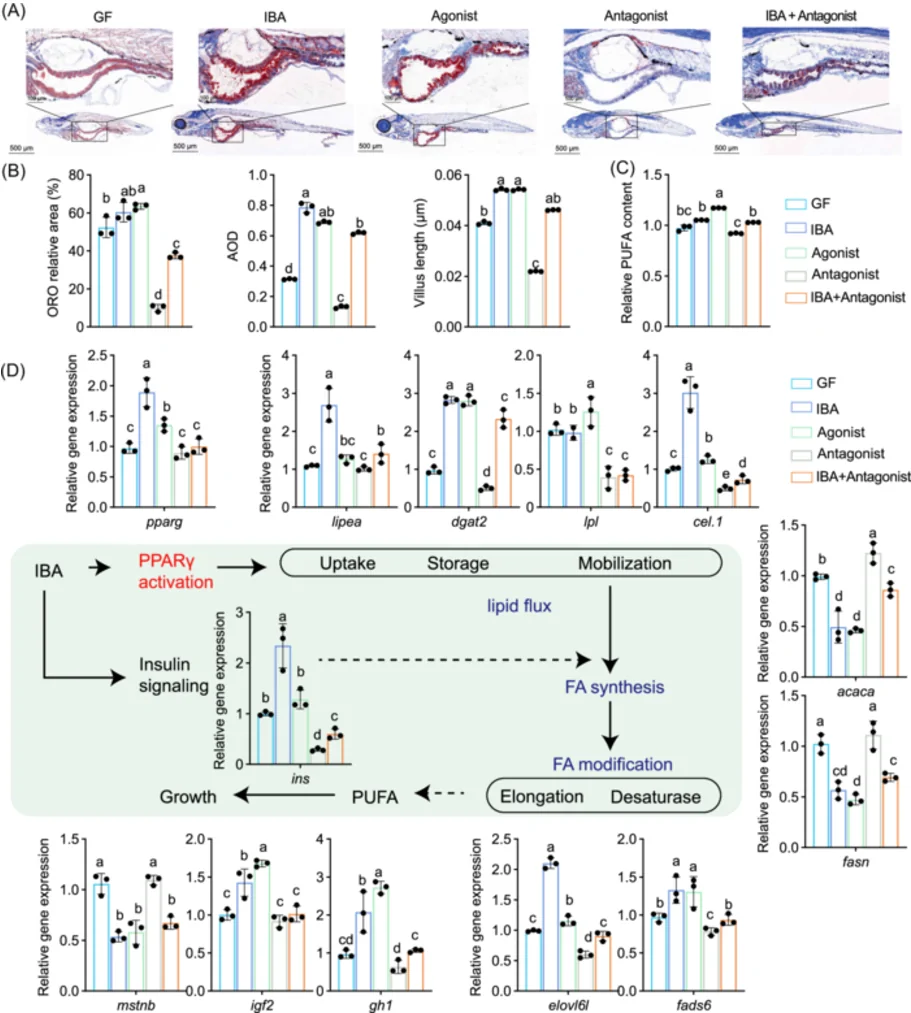
Indole‐3‐butyric acid (IBA) exhibits similar effects on lipid accumulation and PUFA production as PPARγ agonists. (A) Oil Red O (ORO) staining of zebrafish under GF (germ‐free), IBA (indole‐3‐butyric), Agonist, Antagonist, and IBA + Antagonist (indole‐3‐butyric + Antagonist). The staining of the whole fish on the bottom (scale bar = 500 μm) and a localized enlargement of the intestine on the top (scale bar = 100 μm). (B) Quantification of ORO staining shown as relative stained area, average optical density (AOD), and villus length (one‐way ANOVA). (C) Relative PUFA content in zebrafish among different treatments (one‐way ANOVA). (D) Schematic of IBA‐mediated regulatory pathways and gene expression profiles across treatment groups (one‐way ANOVA, *n* = 3 per group). IBA regulates lipid metabolism and growth via two key axes: PPARγ (peroxisome proliferator‐activated receptor gamma) and insulin signaling. These pathways coordinate to modulate lipid flux (blue), fatty acid (FA) synthesis, and FA modification, ultimately linking to growth regulation. Upstream regulators: *pparγ* (PPARγ) and *ins* (insulin). Lipid flux genes: *lpl* (uptake), *dgat2* (storage), *lipea* and *cel.1* (mobilization). FA synthesis genes: *acaca* and *fasn*. FA modification genes: *elovl6l* and *fads6*. Growth‐related genes: *mstnb*, *gh1*, and *igf2*. Different lowercase letters indicate significant differences (*p* < 0.05), *n* = 3 per group.

### Chronic IBA supplementation promotes weight gain and PUFA accumulation in conventional zebrafish

Supplementation with IBA for 4 weeks significantly promoted the growth of juvenile zebrafish, as indicated by elevated relative body weight (*p* < 0.05), whereas body length remained unaffected (Figure 6A). Histological analysis revealed tissue‐specific changes in neutral fat (TAG) deposition, rather than induced overall lipid accumulation (Figure 6B). IBA markedly increased hepatic lipid accumulation, as indicated by both the relative area and the staining intensity of ORO (*p* < 0.05). In contrast, intestinal lipid accumulation showed no significant difference (*p* > 0.05), and muscle deposition was markedly reduced (*p* < 0.05, Figure 6C). Targeted metabolomics identified 24 medium‐ and long‐chain fatty acids (MLCFAs) in muscle tissue. As shown in Figure S7A, PCA was performed to evaluate metabolic separation between groups with PC1 explaining 94.80% of the total variance. Among the detected metabolites, 24 showed upregulated trend (Figure S7B) and docosahexaenoate (DHA) emerged as the only significantly enriched metabolite contributing strongly to group separation (variable importance in projection (VIP) = 1.7, Figure 6D). VIP values of Eicosapentaenoate (EPA), alpha Linolenate (ALA), and Linoleate (LA) were also greater than one. KEGG enrichment analysis revealed that these metabolites were mainly involved in biosynthesis of unsaturated fatty acids, fatty acid biosynthesis, and linoleic acid metabolism (Figure 6E). Consistent with the metabolic data, *pparγ* expression showed an increasing trend, accompanied by significant upregulation of downstream lipid metabolic genes (*dgat2*, *fads6*, *elovl6l*), while the growth‐inhibitory gene *mstnb* was suppressed (*p* > 0.05, Figure 6F). These observations indicated that chronic IBA exposure promoted growth and shifted lipid metabolism toward muscle PUFA accumulation.

**Figure 6:**
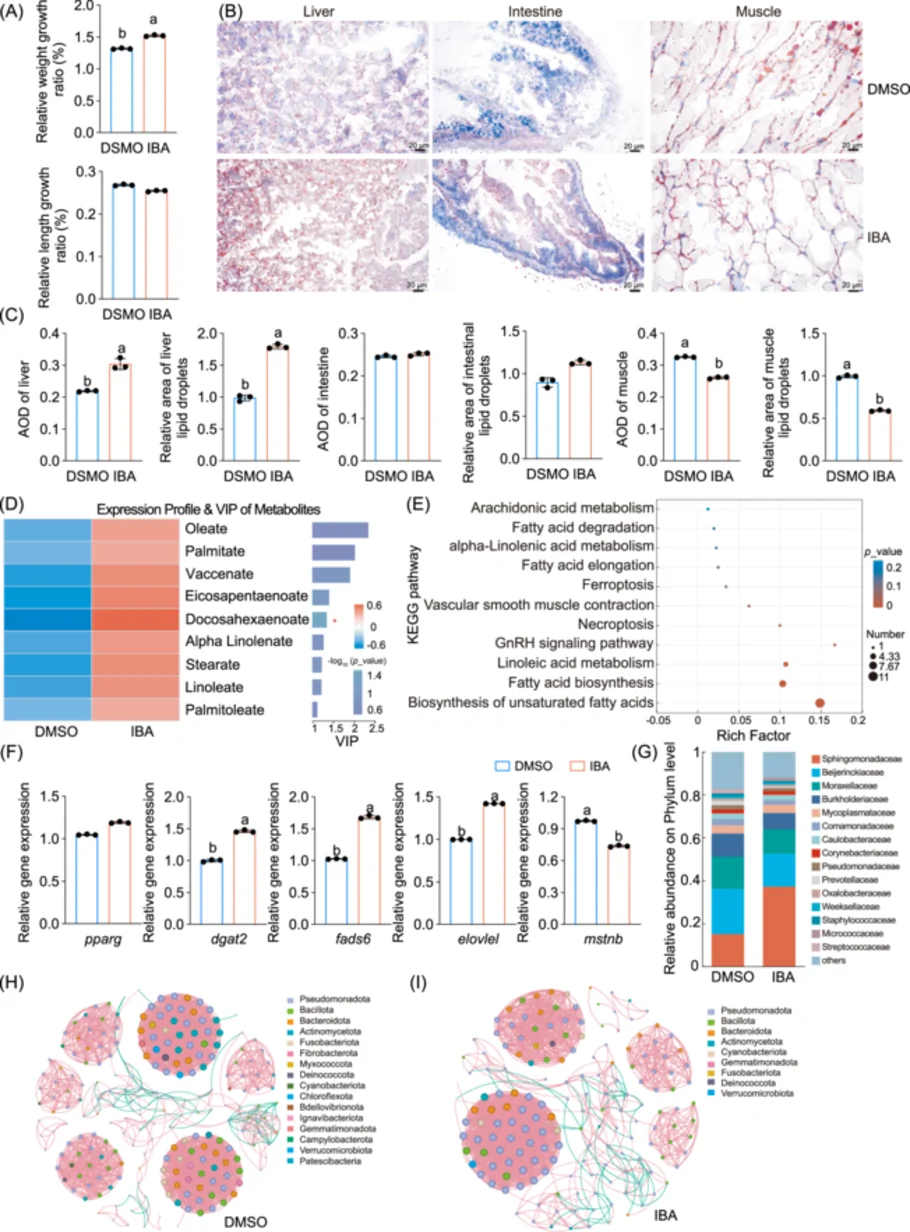
Chronic IBA supplementation promotes growth and lipid metabolism and alters intestinal microbial community composition and interactions. (A) Relative body weight and body length growth rates of zebrafish after 30 days of DMSO or IBA supplementation (*n* = 3 per group). (B) Liver Oil Red O (ORO) staining under DMSO and IBA. Intestinal ORO staining under DMSO and IBA. Muscle ORO staining under DMSO and IBA. Scale bar = 20 μm. (C) Quantification of ORO staining showing the relative stained area and average optical density (AOD) for liver, intestine, and muscle (*n* = 3 per group). (D) Partial Least‐Squares Discriminant Analysis (PLS‐DA) for the comparison groups DMSO versus IBA (VIP > 1). (E) KEGG enrichment of 23 up‐regulated metabolites between the comparison groups. Each bubble in the plot represents a metabolic pathway, and bubble size indicates the number of genes enriched in the pathway (BH‐adjusted *p*‐value). (F) Lipid metabolism‐ and growth‐related genes showed different expression trends in zebrafish muscle under different treatment conditions, including *pparγ*, *dgat2*, *elovl6l*, *fads6* and *mstnb*. Different lowercase letters indicate significant differences (*p* < 0.05). (G) Relative abundance of intestinal microbiota at the family levels in DMSO and IBA groups. Co‐occurrence network analysis shows microbial interaction patterns in DMSO (H) and IBA (I) groups. Nodes in the diagram represent phyla and were classified by degree. The size of each node is the relative abundance. Positive and negative correlations (adjusted *p* < 0.05) are represented by the pink and green lines, respectively.

### IBA modulated gut microbial community composition and function of conventional zebrafish

IBA exposure was also associated with gut microbiota structure and function changes. Significant family‐level alterations were observed, with *Moraxellaceae* significantly enriched and *Burkholderiaceae* reduced in the IBA group (Figure 6G). At the genus level, a total of 85 genera were shared between groups, and Linear discriminant analysis Effect Size (LEfSe) identified *Massilia* as enriched in the DMSO group and *Acidovorax* as a marker for IBA treatment (Figure S7C,D). Alpha‐diversity indices decreased slightly without significance in the IBA group (Shannon, *p* = 0.37; Chao1, *p* = 0.81; Figure S7E). PCA indicated weak separation of microbial communities (PC1 = 33.23%, *R* ^2^ = 0.08, *p* = 0.19; Figure S7F). Integration with targeted metabolomics indicated that the *Burkholderiaceae* abundance negatively correlated with DHA levels, while *Xanthobacteraceae* depletion corresponded with lower *Myristoleate* levels (Figure S7G). Thus, IBA not only affected host lipid biology but also correlated with microbial networks and their metabolic potential. Co‐occurrence network analysis revealed differences in microbial association patterns between the DMSO and IBA groups. Microbial taxa in the DMSO group displayed modular clustering largely by phylum (e.g., *Proteobacteria* and *Bacteroidota*), whereas the IBA group exhibited relatively denser cross‐phylum associations (adjusted *p* < 0.05, Figure 6H,I). Functional prediction (PICRUSt2/Tax4Fun) revealed no significant upregulation of major metabolic pathways between the IBA and DMSO groups (Figure S7H,I).

## 3 DISCUSSION

Marine fish exhibit exceptionally high PUFA levels despite their limited endogenous biosynthetic capacity, a paradox in aquatic lipid metabolism [24]. Through integrative microbiome–metabolome analyses in *S. dumerili*, we identified that gut commensals, particularly Sd_h10, actively participated in host lipid regulation via production of IBA. Functionally, IBA acts as an indole‐derived metabolite potentially involved in PPARγ‐associated regulation, and was correlated with lipid accumulation and PUFA biosynthesis while reshaping intestinal microbial networks. These findings suggest a microbiota‐indole derivatives‐nuclear receptor axis, which may provide additional evidence supporting microbiota‐associated regulation of host lipid metabolism.

The intestinal microbiota has been increasingly recognized as a critical contributor to lipid digestion, absorption, and storage in aquatic animals [25, 26]. Consistent with findings in other fish species [9, 27], the microbiota of *S. dumerili* was highly enriched in lipid metabolism‐related functions, suggesting that intestinal microbes may provide an additional layer of control over host lipid utilization [8, 28]. Microbial lipase, especially from *Bacillus spp*., contributes to the generation of free fatty acids available for host uptake, which has also been observed in other species (*Oreochromis niloticus* and *Pelteobagrus fulvidraco*) [29]. Beyond lipase, the intestinal microbiota also produced diverse lipid‐like and indole derivatives closely correlated with host PUFA biosynthetic pathways, highlighting bacterial taxa, such as *Bacillus* and *Comamonas*. Functional variations in lipid metabolic pathways of the gut microbiota were observed between high‐PUFA and low‐PUFA fish, with lower abundance of fatty acid biosynthesis, biosynthesis of unsaturated fatty acids, secondary bile acid biosynthesis, linoleic acid metabolism, and primary bile acid biosynthesis pathways in low‐PUFA fish (represented by *Oreochromis niloticus*) (Figure S8A) [22, 30, 31, 32, 33]. This pattern aligns with earlier evidence that bacterial metabolites, rather than microbial abundance alone, may contribute to host metabolic outcomes [34, 35]. Collectively, the intestinal microbiota appears functionally adapted to sustain lipid turnover and PUFA enrichment.

Microbial tryptophan metabolism generates a variety of indole derivatives acting as interkingdom signaling molecules [36, 37]. Several of these compounds, such as IAA, indole‐3‐propionic acid (IPA), and indole‐lactic acid, have been shown to influence host glucose and lipid homeostasis via receptor‐mediated signaling [38]. In fish, emerging evidence suggested that microbial indole metabolites may be associated with PUFA metabolism by modulating transcriptional programs controlling fatty acid desaturation and elongation (f*ads*/*elovl*), partly via PPAR and AHR signaling [39]. In this context, IBA was identified in *S. dumerili* may represent a functionally relevant indole derivative linking microbial tryptophan metabolism to host lipid regulation, with potential implications for PUFA‐associated metabolic processes in teleosts with diverse lipid biosynthetic capacities. Although IBA has been less examined in vertebrates, previous studies have reported that it may be involved in modulating metabolic and oxidative stress‐related processes in both mammals and teleosts, despite the absence of defined molecular mechanisms [40, 41, 42, 43]. These observations are consistent with findings that indole derivatives can regulate lipid absorption and bile acid turnover [44], and enhance fatty acid oxidation through PPAR‐related pathways [45]. Together, these results suggest that IBA may serve as a microbial metabolite potentially linking gut microbial activity to host lipid metabolic regulation, including PUFA‐related processes.

A key finding emerging from this study is that the microbiota‐derived metabolite IBA may participate in the modulation of PPARγ‐associated signaling pathways involved in host lipid metabolic regulation. While indole derivatives are commonly linked to AHR‐associated signaling [44, 46], evidence supporting interactions between microbiota‐derived indole metabolites and PPARγ‐related pathways remains comparatively limited, particularly for IBA. In the present study, molecular docking, SPR analysis, PPARγ immunofluorescence assay, and PPARγ downstream gene modulation collectively supported a potential interaction between IBA and PPARγ‐associated signaling. These findings suggest that microbiota‐derived indole metabolites may participate in host lipid metabolic remodeling through nuclear receptor‐associated regulatory pathways in fish.

The biological implications of this interaction are particularly relevant for marine fish, which exhibit high PUFA levels despite limited endogenous desaturation‐elongation capacity [3]. IBA‐associated modulation of PPARγ signaling may contribute to enhanced PUFA‐associated metabolic remodeling [47, 48]. PPARγ governs a conserved transcriptional program that promotes lipid uptake (e.g., *lpl*), esterification and triglyceride assembly (e.g., *dgat2*, *lipea*), and the elongation‐desaturation machinery essential for PUFA production (e.g., *fads6*, *elovl*) [16]. Consistently, IBA exposure reproduced these canonical signatures, aligning with previous reports of PPARγ‐driven lipid partitioning in teleosts [49]. At the organismal level, prolonged IBA treatment promoted hepatic triglyceride accumulation while increasing PUFA deposition in muscle, reflecting the physiological strategy of marine fish to concentrate highly unsaturated fatty acids in peripheral tissues (Figure S8B). In comparison, freshwater fish and marine fish *Siganus canaliculatus*, possess substantial PUFA biosynthetic capacity but exhibit lower muscle PUFA levels, suggesting that both enzymatic potential and bacterial metabolite‐driven signaling influence tissue‐specific PUFA deposition [50]. Notably, long‐chain PUFAs, including DHA and electrophilic derivatives of linoleic acid, could weakly modulate PPARγ themselves [51, 52], raising the possibility that IBA‐induced PUFA accumulation may further enhance PPARγ signaling. Therefore, whether PUFA accumulation secondarily influences PPARγ‐associated signaling requires further investigation. In addition, PPARγ regulation by IBA was accompanied by increased intestinal villus length, a form of intestinal adaptation previously reported for other bioactive indole derivatives and commonly associated with enhanced absorptive capacity [53, 54].

Importantly, IBA supplementation altered the gut community structure, reducing the abundance of these bacterial families, such as *Burkholderiaceae* and *Xanthobacteraceae*, while enhancing fatty acid‐related metabolic potential. However, such shifts were unlikely to be driven solely by direct microbial sensing of IBA. Instead, accumulating evidence suggests a microbiota‐host‐metabolite feedback regulatory loop, in which host metabolic remodeling secondarily reshapes microbial ecology [24, 55, 56, 57]. Consistent with this model, the IBA‐induced reduction of specific bacterial groups correlated with host lipid remodeling, such as increased DHA and reduced myristoleate levels, phenotypes previously shown to modify gut microbial structure via changes in luminal lipid and bile acid environments [58, 59]. Thus, the observed microbial shifts likely reflect a feedback response to IBA‐mediated host lipid reprogramming rather than a purely microbe‐targeted effect.

Several limitations of the present study should be acknowledged. Zebrafish were adopted as a surrogate mechanistic model owing to its well‐established germ‐free culture system, clear genetic background, and conserved lipid metabolic machinery among teleosts. Given the current technical difficulty in constructing germ‐free models for marine *S. dumerili*, zebrafish represented a practical alternative for pathway validation; nevertheless, interspecific differences in lipid regulation and environmental adaptation between the two fish species cannot be ignored. In addition, this work primarily focused on a single bacterial strain and its dominant metabolite IBA, while potential synergistic or antagonistic interactions among multiple microbial metabolites within the complex gut ecosystem remained unclarified. Furthermore, long‐term physiological consequences of continuous IBA or strain intervention, including impacts on growth and reproductive performance of fish, require further systematic evaluation in future aquaculture‐relevant trials.

## 4 CONCLUSION

In conclusion, the present study suggests that intestinal microbiota may participate in PUFA‐associated metabolic remodeling in *S. dumerili* through microbiota–host metabolic interactions. Our findings further support a potential role for the bacterial metabolite IBA in modulating PPARγ‐associated signaling pathways linked to lipid accumulation and PUFA enrichment. These results expand current understanding of microbiota‐associated lipid metabolic regulation in marine fish and provide additional evidence that indole‐derived metabolites may function as important microbiota‐host signaling molecules in vertebrate lipid metabolism. From an aquaculture perspective, modulation of indole‐producing microbiota or microbiota‐derived metabolites might represent a strategy for improving lipid nutritional quality in farmed fish species, although further validation across different teleosts and long‐term evaluations are still required.

## 5 METHODS

### *S. dumerili* intestinal sampling collection

Four healthy 1‐year‐old *S. dumerili* were randomly selected from the culture tanks after fasting for 48 h. Fish were anesthetized with eugenol and disinfected surface with 75% ethanol. The entire intestine was aseptically dissected and divided into foregut (FG), midgut (MG), and hindgut (HG). Intestinal segments were frozen in liquid nitrogen and stored at −80°C until use. Homogenized intestinal tissues were equally subdivided for 16S rRNA sequencing, metabolomic analysis, and bacterial isolation. Additional intestinal samples were rinsed with sterile saline and fixed in 4% paraformaldehyde for histological examination.

### Sd_h10 colonization experiments

To assess the safety of Sd_h10 exposure, 270 healthy 6‐day post‐fertilization (6 dpf) zebrafish were randomly assigned to six groups, each group consisting of 3 tanks (15 fish per tank; *n* = 3). The control group (NC) received sterile physiological saline, while the other groups were exposed to Sd_h10 at concentrations of 1 × 10^2^, 1 × 10^3^, 1 × 10^4^, 1 × 10^5^, and 1 × 10^6^ CFU/mL, respectively. Fish were continuously maintained at 28°C under standard conditions for 10 days, after which survival rates were recorded.

To evaluate the influence of Sd_h10 on host growth and development, zebrafish juveniles (body weight, 0.3 ± 0.09 g) were fed under three experimental conditions: (1) normal diet (artificial feed, NC), (2) high‐fat diet (artificial feed + 2% fish oil, HFD), and (3) HFD with Sd_h10 supplementation (HFD + Sd_h10). Fish were randomly allocated into three groups with 3 replicates (15 fish per tank) and fed twice daily for 14 days. At the end of the feeding trial, relative weight growth rate and relative length growth rate were measured and calculated as follows:

Relative weight growth rate (%) = 100 × (final weight − initial weight)/initial weight

Relative length growth rate (%) = 100 × (final length − initial length)/initial length

For germ‐free (GF) colonization assays, GF and conventionally raised zebrafish were obtained from the same batch of fertilized eggs. GF zebrafish were generated following the sterile hatching procedure described in reported protocols [60]. Briefly, fertilized eggs were disinfected sequentially with 0.003% sodium hypochlorite and 0.05% povidone‐iodine (PVP‐I), rinsed several times with sterile rearing medium (3.5 g NaCl, 0.05 g KCl, 0.025 g NaHCO_3_, 0.1 g/L CaCl_2_), and incubated at 28°C under axenic conditions. Sterility was confirmed by plating rearing medium and homogenized larvae on tryptic soy agar (TSA) and Luria–Bertani (LB) agar at 28°C for 48 h, with no colony growth observed [61]. Larvae at 6 dpf (days post fertilization) were divided into three groups with 3 replicates (15 fish per tank): (1) WT, conventionally cultured zebrafish; (2) GF + Sd_h10, germ‐free zebrafish exposed to Sd_h10 (1 × 10^4^ CFU/mL); and (3) GF, germ‐free zebrafish maintained under sterile conditions. After 24 h of bacterial exposure, zebrafish were fed sufficient finely ground egg yolk. Whole larvae were collected 5 h post‐feeding for transcriptome analysis and ORO staining.

### IBA exposure and feeding experiments

To evaluate the potential toxicity of IBA (Sigma‐Aldrich, 99%) exposure, 60 GF zebrafish larvae (6 dpf) were randomly assigned to two groups with 3 replicates (10 fish per tank): IBA and GF group. IBA was added to culture water at 0.2 μmol/mL, while the GF group received an equal volume of saline. All fish were maintained at 28°C for 10 days, after which survival rates were recorded.

To determine the short‐term metabolic effects of IBA and its interaction with PPARγ signaling, GF zebrafish (6 dpf) were exposed to IBA and pharmacological modulators of PPARγ. Sterile egg yolk suspension was prepared by mixing the sterile egg yolk with sterile water at a ratio of 1:10 (w/w) and served as the feeding matrix. IBA, the PPARγ agonist rosiglitazone, and the antagonist GW9662 were separately mixed into yolk (20 μmol/mL final concentration) and diluted to 0.2 μmol/mL in aquatic water. GF zebrafish were randomly allocated to five groups with 3 replicates (20 fish per tank): GF, IBA, agonist, antagonist, and IBA + antagonist. Whole larvae were collected 5 h post‐feeding for ORO staining, PUFA quantification (ELISA, Huabang Biotechnology), and quantitative real‐time PCR (qRT‐PCR).

To further investigate the chronic effects of IBA on growth, lipid deposition, and gut homeostasis, 60 zebrafish (initial body weight 0.28 ± 0.03 g, length 1.8 ± 0.2 cm) were randomly divided into two groups with 3 replicates (10 fish per tank). The control group was exposed to water containing <0.1% dimethyl sulfoxide (DMSO). DMSO was used as the solvent to prepare the IBA working solution, and the treatment group was supplemented with 2 μmol/mL IBA (Sigma‐Aldrich, ≥ 99%). Water was renewed every 2 days. After 30 days, fish were euthanized with ice and surface‐disinfected with 75% ethanol. Muscle samples were frozen in liquid nitrogen and collected for targeted metabolomic analysis and qRT‐PCR. Intestinal tissues were equally divided for 16S rRNA sequencing, while muscle, intestine, and liver tissues were fixed for ORO staining.

### 16S rRNA sequencing and data analysis

All 16S rRNA sequencing procedures were conducted following protocols reported in previous studies [62, 63]. Genomic DNA was extracted from intestinal contents using the HiPure Stool DNA Kit (Magen). The hypervariable V3–V4 region of the bacterial 16S rRNA gene was amplified with the primer pair 341F/805R. Library preparation and paired‐end sequencing were performed on an Illumina NovaSeq. 6000 platform. Raw sequencing reads were processed using the UPARSE pipeline (v9.2.64) [64] to cluster high‐quality sequences into OTUs at a 97% similarity threshold. Alpha diversity indices were calculated with QIIME (v1.9.1) [65] and compared between groups using Welch's *t*‐test. Beta diversity was assessed via PCA based on Bray–Curtis distances. LEfSe [66] was employed to identify differentially abundant taxa across groups (*q* < 0.05 and LDA score > 3). For functional prediction, the phylogenetic investigation of communities by reconstruction of unobserved states (PICRUSt2, v2.1.4) [67] was used to infer Kyoto Encyclopedia of Genes and Genomes (KEGG) pathways [68]. Additional functional profiling was conducted using Tax4Fun. All statistical analyses and visualizations were performed using the online Omicsmart analytical tool (http://www.omicsmart.com). Pairwise associations among bacterial genera were evaluated using Spearman's rank correlation analysis implemented in the R package psych. To account for multiple testing, *p*‐values were adjusted using the Benjamini–Hochberg false discovery rate (BH‐FDR) procedure. Correlations with BH‐adjusted *p* < 0.05 were considered statistically significant.

### Metabolomics analysis

Untargeted metabolomic profiling was performed to comprehensively characterize the intestine of *S. dumerili*. Intestinal tissues were defrosted at 4°C, and the extraction of metabolites and LC‐MS metabolomics analysis were performed following the protocol described by Yuan et al. [69]. Untargeted metabolomic analysis was performed using an Agilent 1290 Infinity UHPLC system (Agilent) coupled with an AB SCIEX Triple TOF 6600 mass spectrometer (AB SCIEX). Metabolite separation was carried out using a Waters ACQUITY UPLC BEH Amide column (2.1 mm × 100 mm, 1.7 μm) in HILIC mode. The column temperature was 25°C, flow rate was 0.5 mL/min, and injection volume was 2 μL. Mobile phase A consisted of water containing 25 mM ammonium acetate and 25 mM ammonium hydroxide, and mobile phase B was acetonitrile. The gradient elution program was set as follows: 95% B at 0–0.5 min; linear decrease to 65% B at 0.5–7 min; linear decrease to 40% B at 7–8 min; 40% B maintained at 8–9 min; rapid return to 95% B at 9.1 min, followed by equilibration until 12 min. Electrospray ionization (ESI) in both positive and negative ion modes was used. The IonSpray Voltage Floating was set to ±5500 V. QC samples were prepared by mixing equal volumes of all experimental samples. QC samples were analyzed regularly before, during, and after analytical batches, and inserted every five samples to monitor system stability, precision, and data reliability throughout acquisition. Data normalization was performed based on QC sample‐based signal correction and internal standard calibration to ensure accuracy and repeatability of metabolite quantification. Raw data were processed using Progenesis QI (v3.0) for peak picking, alignment, and normalization. Metabolites were identified by matching MS and MS/MS spectra against reference databases with a mass tolerance of <10 ppm.

Targeted metabolomic profiling was performed on zebrafish muscle. Samples (50 mg, *n* = 6 per group) were homogenized, ultrasonicated, centrifuged, and the resulting supernatant was dried under nitrogen. Fatty acid methyl esters were derived by reaction with 0.5 M NaOH‐methanol at 60°C for 30 min and extracted into n‐hexane for gas chromatography‐mass spectrometry (GC‐MS) analysis. GC‑MS analysis was performed using an Agilent 8890B system coupled with an Agilent 5977B/7000D mass spectrometer (Agilent). Separation was carried out on an Agilent CP‑Sil88 for FAME column (100 m × 0.25 mm × 0.20 µm) with helium (99.999%) as the carrier gas at a constant flow rate of 1 mL/min. The oven temperature program was as follows: held at 80°C, increased to 180°C at 8°C/min, raised to 220°C at 4°C/min, further increased to 230°C at 2°C/min and held for 13.5 min, and finally maintained at 235°C for 2 min. The injection volume was 1 μL with a split ratio of 50:1, and the inlet temperature was 260°C. Electron impact (EI) ionization was used at 70 eV. The ion source temperature was 230°C, and the quadrupole temperature was 150°C. Detection was performed in selected ion monitoring (SIM) mode. Quality control (QC) samples were prepared by pooling all experimental samples and inserted every 5–15 samples to monitor system stability and analytical repeatability. Quantification was achieved using external calibration curves (*R* ^2^ > 0.99).

Differential metabolites were identified using variable importance in projection (VIP ≥ 1) and *p*‐value results. KEGG‐based pathway enrichment analysis was performed for differential metabolites (BH‐adjusted *p*‐value). Sequencing and bioinformatic analyses were completed with the online Omicsmart analytical tool (http://www.omicsmart.com).

### Isolation, identification, and characterization of commensal strains

Homogenized *S. dumerili* foregut and hindgut samples were serially diluted with sterile saline and spread onto marine 2216E, LB, and TCBS agar plates under aerobic conditions. A 2.5 L vertical round‐bottom anaerobic cultivation bag (HOPEBIO) was used to establish an anaerobic environment for anaerobic microbial isolation and cultivation. No additional supplements were added to the culture media. Single colonies with distinct morphological traits (size, edge, surface texture, and pigmentation) were picked after incubation at 28°C for 24–48 h, then purified via three rounds of streak‐plate subculture to acquire pure isolates. 16S rDNA was amplified with universal primers 27‐F and 1492‐R (Table S5). PCR products were sequenced by BGI Genomics (Shenzhen, China), and taxonomic identification was conducted via BLAST search against the NCBI database (https://blast.ncbi.nlm.nih.gov/Blast.cgi). Duplicates were removed and appropriate sequences were selected for the phylogenetic tree, which was constructed by the neighbor‐joining method [70] using the software MEGA‐X 10.2.2 (https://www.megasoftware.net/) [71]. Bacterial strains were further characterized by Gram staining.

Antibiotic susceptibility was assessed against 20 antibiotics (TET, GEN, PB, PEN, AMP, E, CAZ, CZ, MI, AMK, CPZ, CN, DO, S, PIP, CTR, VAN, CXM, KAN, MY; A comprehensive list of all abbreviations is available in Table S6) using the paper diffusion method. Hemolytic activity was evaluated by inoculating 20 μL of overnight bacterial cultures onto ready‐to‐use blood agar plates (Huankai Microbial). Plates were incubated at 28°C for 48 h, and hemolytic zones were recorded.

### Extracellular enzyme activity assay

Twelve bacterial isolates belonging to eight different families were selected based on their hemolytic capacity. To prepare extracellular protein extracts, isolates were cultured on TSA agar overlaid with sterile cellophane at 28°C for 48 h. Bacterial lawns were harvested, and proteins were extracted with 1× phosphate‐buffered saline (PBS, pH = 7.2). The resulting crude extracts were filter‐sterilized and stored at −20°C until use. Enzyme activities were assessed using plate‐based assays according to established methods [72]. Briefly, 20 µL of each extract was loaded into wells on agar plates containing specific substrates to detect protease, containing 1% gelatin, 1% casein, 1% starch, 1% Tween 80, 2% urea (with 0.5% phenol red indicator), 8% skim milk, or 2.5% egg yolk. Plates were incubated at 28°C for 12 h. The appearance of hydrolysis or a transparent circle indicated positive activity.

### Lipid utilization assessment of candidate strains

Based on enzyme activity profiles, three strains with lipase activity, Sd_y1 (*Niallia alba*), Sd_Y15 (*Pseudomonas yangonensis*), and Sd_h10 (*Bacillus thuringiensis*), were selected for lipid utilization assays. Strains were inoculated into Tryptic Soy Broth (TSB) supplemented with a range of concentrations (v/v, 2–16%) of fish oil, soybean oil, or paraffin. Growth was monitored in a Thermo Scientific™ Multiskan™ GO microplate reader (Thermo Fisher Scientific) at 28°C with shaking. Furthermore, following 18 h of incubation in medium containing fish oil as the sole carbon source, spent culture supernatant of Sd_h10 and Sd_y15 was collected for subsequent untargeted metabolomic analysis via LC–MS to characterize lipid‐derived metabolites. To further evaluate the lipid utilization capacity of Sd_h10, basal medium with 2% fish oil as the sole carbon source was used. Levels of triglycerides (TG) and free fatty acids (FFA) in the aqueous phase were quantified using enzymatic assay kits (Amplex Red, Beyot Beyotime Biotechnology).

### Histological staining

For morphological assessment, fixed intestine samples were processed for hematoxylin and eosin (H&E) staining following the approach of Erben et al. [73]. Villus length and intestinal wall thickness were quantified from five randomly selected sections per group. Neutral lipids in zebrafish tissues were visualized using ORO staining, according to the established protocol [74]. Each biological replicate included more than three sections for quantification. Images were acquired with a light microscope, and lipid‐positive areas were quantified using ImageJ (v1.53f) [75].

### Transcriptome analysis

RNA was extracted, libraries were constructed, and sequencing was conducted following the methodology outlined by Qu et al. [76]. The number of read counts for a gene or transcript was normalized using Transcripts Per Million. Differential expression analysis of RNAs between two distinct groups was carried out using DESeq. 2. Genes with a FDR below 0.05 and an absolute fold change ≥ 2 were identified as differentially expressed genes. Pathway enrichment analysis based on the KEGG was performed using KOBAS (http://kobas.cbi.pku.edu.cn/home.do) [77]. Bioinformatic analyses were completed with the online Omicsmart analytical tool (http://www.omicsmart.com).

### qRT‐PCR

qRT‐PCR was performed following the protocol of Qu et al. [78]. Primers used for qRT‐PCR are listed in Table S5. The relative expression of target genes was calculated using the 2^−ΔΔCt^ method with β‐actin as an internal reference. Details of the qRT‐PCR reaction system and amplification procedure are shown in Tables S7 and S8, respectively.

### Molecular docking

The zebrafish pparγ gene sequence was retrieved from NCBI (https://www.ncbi.nlm.nih.gov/), and the amino acid sequence of the zebrafish PPARγ was obtained from the UniProt database (https://www.uniprot.org/) [79]. The structure of the protein was predicted using AlphaFold 3 (https://alphafoldserver.com/) [80] and saved in PDB format. Ligand structure of IBA was obtained from ChemSpider (https://www.chemspider.com/) in MOL format. Protein and ligand structures were prepared using AutoDock Tools. Polar hydrogens were added to the PPARγ receptor, and Gasteiger charges were calculated. A 3D affinity grid box was generated to cover the potential ligand‐binding pocket, with a grid spacing of 0.375 Å. Molecular docking was performed using AutoDock 4.2.6 (https://autodock.scripps.edu/) [81] with the Lamarckian genetic algorithm to calculate the binding affinity between PPARγ and IBA. The best docking conformation was selected based on the lowest binding energy (kcal/mol), where a lower value indicates stronger and more stable binding. All docking results were visualized, analyzed, and processed using PyMOL (https://pymol.org/).

### Expression and purification of PPARγ protein

The zebrafish PPARγ gene was synthesized and cloned into the pCZN1 expression vector using NdeI/XbaI restriction sites, generating a fusion protein with an N‐terminal 6×His tag. The recombinant plasmid was transformed into *Escherichia coli* ArcticExpress (DE3) cells for heterologous expression. A single colony was inoculated into LB medium containing Ampicillin (Amp) and cultured at 37°C to mid‐log phase. Protein expression was induced with 0.2 mM Isopropyl‐beta‐d‐thiogalactopyranoside at 15°C for 4 h [82]. Cells were harvested by centrifugation, resuspended in lysis buffer (20 mM Tris‐HCl, 300 mM NaCl, 10 mM imidazole, pH 8.0), supplemented with protease inhibitors, and lysed by ultrasonication on ice. The soluble fraction was collected by centrifugation and subjected to immobilized metal affinity chromatography using Ni‐NTA resin under native conditions [83]. The purified protein exhibited an apparent molecular mass of ~62.4 kDa and a purity >85% based on SDS‐PAGE analysis, with a final concentration of ~0.5 mg·mL^−1^. Protein identity was verified by Western blot using an anti‐His antibody. Aliquots were stored in PBS (pH 7.4) at −80°C until use.

### Surface plasmon resonance (SPR) binding assay

The interaction between IBA and PPARγ was characterized using a Biacore 8 K instrument (Cytiva) at 25°C. PPARγ was immobilized on a Series S Sensor Chip CM5 (Cytiva, 29149603) via standard amine coupling chemistry using the manufacturer's Amine Coupling Kit (Cytiva, BR100050). A reference flow cell was regulated and blocked identically but without protein immobilization to serve as a blank for nonspecific binding and bulk refractive index subtraction. Serial dilutions of IBA (0–50 µM) were prepared in running buffer (1× PBS‐P+, Cytiva 28995084, supplemented with 5% DMSO and 0.05% Tween‐20, pH 7.4). Analyte solutions were injected over the immobilized PPARγ surface and the reference surface at a flow rate of 30 µL/min with a 60‐second association phase followed by a 60‐second dissociation phase. Solvent correction was performed by including a standard DMSO calibration curve to account for background signals. The sensor surface was regenerated with a 60‐second injection of 10 mM glycine‐HCl (pH 2.0) between analyte cycles.

### Immunofluorescence (IF) staining

IF staining was performed to detect PPARγ nuclear translocation in GF zebrafish larvae. GF zebrafish larvae at 6 dpf (days post fertilization) were used for IF analysis and randomly divided into four groups, four biological replicates (5 fish per tank): (1) GF control; (2) IBA treatment (0.2 μmol/mL); (3) PPARγ agonist (rosiglitazone, 0.2 μmol/mL); (4) PPARγ antagonist (GW9662, 0.2 μmol/mL). DMSO was used as the solvent for all working solutions. Larvae were fed with finely ground egg yolk. After 5 h of exposure, whole larvae were collected and fixed in 4% paraformaldehyde. For immunofluorescence staining, larvae were washed with PBS three times, and then permeabilized with 0.5% Triton X‐100 (Beyotime) for 20 min, rinsed with PBST, and blocked with QuickBlock™ buffer (Beyotime) for 10 min. Samples were incubated with primary antibody against PPARγ (1:1000, Anti‐zebrafish PPAR Gamma/PPARG Antibody, Boster) at 4°C overnight. After washing with PBST, larvae were incubated with Dylight 488, goat anti‐rabbit IgG secondary antibody (1:1000, Abbkine) at room temperature for 1 h in the dark. Nuclei were counterstained with DAPI reagent (SuperKine^TM^ Enhanced Antifade Mounting Medium, Abbkine). Fluorescence images were captured using a confocal laser scanning microscope (Leica SP8). The nuclear localization of PPARγ was quantified by relative fluorescence intensity and Manders' colocalization coefficient (tM1) through Fiji ImageJ (1.54e). Relative fluorescence intensity was measured as the mean fluorescence intensity of PPARγ. tM1 was calculated to quantify the overlap between PPARγ and DAPI signals.

### Integration of microbiome and metabolomics analysis

Metabolites with clear annotation information and species with relative abundance >0.1% were selected for association analysis. Bidirectional orthogonal projections to latent structures (O2PLS) analyses were performed using the OmicsPLS package based on metabolite abundance and species abundance of microorganisms [84]. Bioinformatic analyses were completed with the online Omicsmart analytical tool (http://www.omicsmart.com).

### Statistical analysis

Each biological replicate was defined as an independent tank of fish. All measurements from individual fish or microscopic fields within the same tank were averaged prior to statistical analysis. Data were presented as mean ± standard deviation (SD). Normality and homogeneity of variance were assessed using Shapiro–Wilk and Levene's tests. For comparisons between two groups, two‐tailed Student's *t*‐test was applied. Specifically, *t‐*test was used to compare TG and FFA levels (0 h vs. 36 h), growth‐related indices, ORO measurements (relative stained area and average optical density), villus length, and gene expression differences (*pparγ*, *dgat2*, *elovl6l*, *fads6*, and *mstnb*) between DMSO and IBA groups. For multiple‐group comparisons among NC, HFD, and HFD + Sd_h10 groups, one‐way ANOVA followed by Tukey's post hoc test was used. Similarly, one‐way ANOVA was applied to analyze Manders' tM1 coefficient and relative fluorescence intensity. Tukey's honestly significant difference test was used for multiple comparisons. Statistical significance was defined as *p* < 0.05. Two‐way analysis of variance (ANOVA) was used to evaluate the effects of IBA, antagonist, and their interaction. When significant effects were detected, post hoc comparisons were performed. Effect sizes were calculated as partial eta squared, and 95% confidence intervals were estimated for interaction effects.

## AUTHOR CONTRIBUTIONS

**Ruojing Li**: Methodology; validation; visualization; writing—review and editing. **Biao Yuan**: Writing—original draft; visualization. **Xin Yi**: Methodology; writing—review. **Jieyan Lin**: Validation; investigation. **Bi Huang**: Writing—review. **Ermei Yin**: Validation; investigation. **Jiayu Zhou**: Methodology; investigation. **Qianwen Min**: Methodology; investigation. **Meiqi Lv**: Methodology; investigation. **Bo Zhang**: Conceptualization; writing—review and editing; supervision; project administration; funding acquisition. **Na Zhao**: Formal analysis; methodology; investigation; resources; funding acquisition.

## CONFLICT OF INTEREST STATEMENT

The authors declare no conflicts of interest.

## ETHICS STATEMENT

The ethics application (No. 202307081) was approved by the Research Ethics Committee of the Southern Marine Science and Engineering Guangdong Laboratory (Zhanjiang).

## AI STATEMENT

The authors acknowledge the use of GPT‑4o (OpenAI) to polish English expression, improve sentence fluency, and grammar of the manuscript. All outputs have been verified for accuracy by the authors.

## Supporting information


**Figure S1**: Gut microbiota composition and functional profiling in *S. dumerili* intestinal segments.
**Figure S2**: Metabolomic profiling and pathway enrichment in *S. dumerili* intestine.
**Figure S3**: Microbial‐metabolite interactions linked to lipid metabolism.
**Figure S4**: Growth curves of three isolated bacteria.
**Figure S5**: Analysis of zebrafish transcriptome data and security of IBA.
**Figure S6**: Effects of IBA and antagonist treatments on lipid metabolism and gene expression assessed by two‐way ANOVA.
**Figure S7**: IBA exposure induced gut microbiota structure and function changes.
**Figure S8**: Microbiota and IBA contribute to lipid accumulation and PUFA production in fish.
**Table S1**: Primer Sequences.
**Table S2**: Alpha diversity index table.
**Table S3**: Endemic and core species in each comparison group (genus level).
**Table S4**: Two‐way ANOVA results for IBA and antagonist treatments.
**Table S5**: Circle of inhibition diameter of isolated bacteria under antibiotics (cm).
**Table S6**: Comprehensive list of all antibiotic abbreviations.
**Table S7**: qRT‐PCR reaction system.
**Table S8**: qRT‐PCR reaction procedure.

## Data Availability

The data that support the findings of this study are available from the corresponding author upon reasonable request. All the sequencing data have been deposited in NCBI and NGDC under submission numbers PRJNA1004436 (https://www.ncbi.nlm.nih.gov/bioproject/PRJNA1004436), PRJNA1192734 (https://www.ncbi.nlm.nih.gov/bioproject/PRJNA1192734), and PRJCA058101 (https://ngdc.cncb.ac.cn/bioproject/browse/PRJCA058101). Metabolomics data are available in MetaboLights MTBLS13872 (https://www.ebi.ac.uk/metabolights/MTBLS13872) and OMIX under submission number OMIX015114 (https://ngdc.cncb.ac.cn/omix/release/OMIX015114). The data and scripts used are saved in GitHub https://github.com/Ermao93/Li2026imetaomics. Supplementary materials (figures, tables, graphical abstract, slides, videos, Chinese translated version, and updated materials) may be found in the online DOI or iMeta Science http://www.imeta.science/imetaomics/.
