# Supplementary material for: Bacterial indole‐3‐butyric acid promotes fish PUFA synthesis
Source: IMetaOmics. 2026 Aug 26;3(3):e70136. doi: 10.1002/imo2.70136 (PMC13674406; doi:10.1002/imo2.70136)
Supplement: Supplementary file 1 [file IMO2-3-e70136-s001.docx]

**Supporting information to**

**Bacterial indole-3-butyric acid promotes fish PUFA synthesis**

**Running title:** IBA regulates PPARγ to boost PUFA synthesis in fish

Ruojing Li^#^, Biao Yuan ^#^, Xin Yi, Jieyan Lin, Bi Huang, Ermei Yin, Jiayu Zhou, Qianwen Min, Meiqi Lv, Bo Zhang^*^, Na Zhao^*^

Southern Marine Science and Engineering Guangdong Laboratory (Zhanjiang), Zhanjiang, 524000, China

^#^These authors contributed equally: Ruojing Li, Biao Yuan

* Correspondence: zb611273@163.com (Bo Zhang); penguinzn@163.com (Na Zhao)

**FIGURE S1: Gut microbiota composition and functional profiling in *S. dumerili* intestinal segments.**

(A) Anosim test of microbiota structure at OTU level across foregut (FG), midgut (MG), and hindgut (HG) (*p* = 0.026). (B) Relative abundance of top 10 phyla in each comparison group. (C) KEGG level 2 functional pathways (top 5), highlighting lipid and amino acid metabolism. (D) Venn diagram of shared and unique genera across segments.

**FIGURE S2: Metabolomic profiling and pathway enrichment in *S. dumerili* intestine.**

(A–C) OPLS-DA score plots of metabolites in FG vs. MG, FG vs. HG, and MG vs. HG (VIP > 1.0, *p* < 0.05). (D) KEGG classification of enriched metabolites (lipid and amino acid metabolism dominant). FG, foregut; MG, midgut; HG, hindgut.

**FIGURE S3: Microbial-metabolite interactions linked to lipid metabolism.**

(A–B) KEGG pathways enriched in downregulated metabolites (FG vs. HG/MG), including vitamin and amino acid metabolism (Qvalue < 0.05). FG, foregut; MG, midgut; HG, hindgut. (C) Downregulated metabolites in MG vs. HG, enriched in carbohydrate and amino acid metabolism (Qvalue < 0.05). (D) Metabolomics pathway analysis (MetPA) showing that differential metabolites shared among FG, MG, and HG were involved in lipid-associated pathways (impact value > 0.3). (E) Two-way orthogonal partial least squares (O2PLS) correlation analysis between gut microbiota and metabolites in *S. dumerili* intestines, among which metabolites such as pterine and linoleic acid had a greater impact on the bacterial community structure in the intestine.

**FIGURE S4: Growth curves of** **three** **isolated bacteria.**

(A–C) Growth curves of *Niallia alba* Sd_y1 in tryptic soy broth (TSB) supplemented with fish oil, soybean oil, or paraffin. The strains show similar growth patterns across all lipid sources. (D–F) Growth curves of *Pseudomonas yangonensis* Sd_Y15 under the same conditions, showing enhanced growth in fish oil. (G–I) Growth curves of *Bacillus thuringiensis* Sd_h10 displayed the highest growth rate and biomass in fish oil compared to soybean oil and paraffin.

**FIGURE S5: Analysis of zebrafish transcriptome data and security of IBA.**

(A) Survival rate of zebrafish exposed to Sd_h10 (1×10²–1×10⁶ CFU/mL; no mortality). (B) PCA of transcriptomes from WT (Wild type), GF (Germ-free), and GF + Sd_h10 (Germ-free + Sd_h10) zebrafish (95% confidence ellipses). (C) Venn diagram of shared differentially expressed genes (DEGs) between experimental groups. (D–E) Validation of RNA-seq data by qRT-PCR (R² = 0.9129). (F) Survival rates of zebrafish larvae (6 dpf) exposed to IBA treatment.

**FIGURE S6: Effects of IBA and antagonist treatments on lipid metabolism and gene expression assessed by two-way ANOVA.**

(A–D) Intestinal lipid accumulation and metabolic indicators including ORO (Oil Red O staining), AOD (average optical density), villus length, and relative PUFA content under GF (Germ-free, control) or IBA (Indole-3-butyric) conditions in the presence or absence of antagonist treatment. (E–H) Relative gene expression levels of lipid metabolism-related genes (*pparγ*, *elovl6l*, *fads6*, and *dgat2*) under GF or IBA conditions in the presence or absence of antagonist treatment. Data are presented as mean ± SD. Statistical differences among groups were analyzed using two-way ANOVA followed by appropriate post hoc comparisons. Different letters indicate significant differences among treatments (*p* < 0.05).

**FIGURE S7: IBA exposure induced gut microbiota structure and function changes.**

(A) Principal component analysis (PCA) for metabolome of DMSO (Dimethyl sulfoxide) and IBA (Indole-3-butyric) groups. (B) Volcano plots of the number of differentially expressed metabolites in DMSO vs. IBA. Gray dots: non-significant MLCFAs (adjusted *p* > 0.05). Dashed line indicates significance threshold (*p* = 0.05). **(**C) Venn diagram shows shared genera between DSMO and IBA groups. (D) Histogram Linear discriminative analysis (LDA) of LEfSe scored greater than 3 presents in DMSO and IBA at the genus level. The color of the histogram represents different groups, and the length represents the LDA value (LDA score > 3.0, FDR < 0.05). (E) Alpha diversity indices for comparison groups (Shannon and Chao1) in the DMSO and IBA groups. (F) PCA (Bray Curtis distance) based on the relative abundance of genus. Ellipses represent 95% confidence intervals. (G) Spearman correlation heatmap between 14 PUFAs and intestinal microbiota at the family level. (H–I) The heatmaps of microbiota function predicted through the PICRUSt2 and Tax4fun databases.

**FIGURE S8: Microbiota and IBA contribute to lipid accumulation and PUFA production in fish.**

(A) Differences in lipid metabolism pathway enrichment results from gut microorganisms of fish, which were collected from published experiment data with different PUFA contents (Top5 pathway) [31–35]. The values in the heatmap were the result of the abundance raw data processed by logarithmic scaling with a base of 10. (B) Hypothesis diagram of the mechanism that IBA produced by Sd_h10 may regulate lipid metabolism in fish through modulation of PPARγ-associated signaling pathways. Red and blue ovals represent up- and down-regulated genes, respectively. Rounded rectangles represent involved KEGG metabolic pathways.
